# Raloxifene as an Adjuvant Therapy for Patients With Schizophrenia: An Up‐To‐Date Systematic Review and Meta‐Analysis

**DOI:** 10.1002/brb3.70649

**Published:** 2025-07-20

**Authors:** Tzu‐Rong Peng, Hung‐Hong Lin, Jian‐Ying Wang, Ming‐Chia Lee, Shih‐Ming Chen

**Affiliations:** ^1^ Department of Pharmacy, Taipei Tzu Chi Hospital Buddhist Tzu Chi Medical Foundation New Taipei City Taiwan; ^2^ School of Pharmacy, College of Pharmacy Taipei Medical University Taipei Taiwan; ^3^ Department of Pharmacy Chia‐Nan University of Pharmacy and Science Tainan Taiwan; ^4^ Department of Pharmacy New Taipei City Hospital New Taipei City Taiwan

**Keywords:** meta‐analysis, psychosis, raloxifene, schizophrenia

## Abstract

**Background and Hypothesis:**

Raloxifene may be useful as an adjunctive treatment for schizophrenia, particularly in addressing psychotic symptoms. This meta‐analysis aimed to evaluate the effectiveness and safety of adjunctive raloxifene in improving positive, negative, and general psychopathology symptoms, as measured by the Positive and Negative Syndrome Scale (PANSS).

**Study Design:**

A systematic search was performed using PubMed, Embase, and the Cochrane Library databases for articles published until May 2024. Randomized controlled trials investigating the effectiveness and safety of adjunctive raloxifene for treating schizophrenia were included. The primary outcome was psychotic symptom severity using PANSS subscales. Mean differences (MDs) and their 95% confidence intervals (CIs) were calculated using random effects models.

**Study Results:**

Ten studies were included in the final analysis. Compared with the placebo group, raloxifene as an adjunctive therapy significantly improved the positive, negative, general, and total PANSS scores, MD = −1.00 (95% CI = −2.00 to −0.20; *I* ^2^ = 48%; *p* = 0.02; *τ* ^2^ = 0.87), MD = −1.35 (95% CI = −2.74 to 0.04; *I* ^2^ = 71%; *p* = 0.06; *τ* ^2^ = 3.27), MD = −3.29 (95% CI = −5.74 to −0.83; *I* ^2^ = 74%; *p* = 0.009; *τ* ^2^ = 9.59), and MD = −7.12 (95% CI = −11.89 to −2.36; *I* ^2^ = 74%; *p* = 0.003; *τ* ^2^ = 41.86), respectively.

**Conclusions:**

Adjunctive raloxifene appears to be a safe and effective treatment for improving positive, general, and total symptoms in patients with schizophrenia, particularly in those with mild‐to‐moderate illness and postmenopausal women. The 60 mg daily dose over at least 12 weeks yielded the most consistent benefits. Further high‐quality trials are needed to confirm its efficacy across diverse populations and guide personalized treatment strategies.

## Graphical abstract

Subgroup analysis by treatment duration (≤12 Weeks): Adjunctive raloxifene demonstrated statistically significant efficacy across all PANSS domains in trials with treatment duration of 12 weeks or less:

**• PANSS Positive symptoms**: MD = −1.48; 95% CI: −2.41 to −0.39

**• PANSS Negative symptoms**: MD = −1.63; 95% CI: −3.15 to −0.12

**• PANSS General psychopathology**: MD = −3.75; 95% CI: −6.34 to −1.16

**• PANSS Total score**: MD = −8.07; 95% CI: −12.7 to −3.44

These findings suggest that short‐term adjunctive treatment with raloxifene (≤12 weeks) may be associated with clinically meaningful improvements in schizophrenia symptoms.

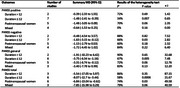

## 1 Background

Schizophrenia is a chronic and severe mental illness that affects approximately 1% of the global population (Tandon et al. 2009). It typically develops in early adulthood and significantly impairs social and occupational functioning. Clinically, schizophrenia is characterized by positive symptoms (e.g., hallucinations and delusions), negative symptoms (e.g., affective flattening and lack of motivation), and cognitive symptoms (e.g., deficits in attention and memory) (Shilling and Feifel 2016; Stepnicki et al. 2018). Although current antipsychotic medications are effective in alleviating positive symptoms, they are less effective for negative and cognitive symptoms. This limitation is especially evident in patients with chronic or treatment‐resistant schizophrenia, who often require multiple antipsychotics, increasing the risk of metabolic side effects (Jeon and Kim 2017). Therefore, identifying new treatment options and adjunctive therapies has become a major focus of clinical and research interest.

Sex differences also play a critical role in schizophrenia. Compared with men, women generally experience a later onset (by 1–5 years) and may have a second peak of incidence around menopause (Mendrek and Mancini‐Marïe 2016; Häfner 2003; Gurvich et al. 2019). Increasing evidence suggests that estrogen may be a key factor contributing to these differences (Mortimer 2007; Kumari 2011; Talonen et al. 2017). Some antipsychotic medications elevate serum prolactin levels, which suppress estrogen secretion, potentially creating a neurochemical environment unfavorable to symptom control (Peuskens et al. 2014). Studies have shown that women with schizophrenia tend to have lower estrogen levels than healthy controls, and disease onset or relapse often coincides with phases of low estrogen during the menstrual cycle (Riecher‐Rössler and Kulkarni 2011). While estradiol has been used as adjunctive treatment for schizophrenia, long‐term use is limited due to its potential adverse effects on breast and endometrial tissue (Kulkarni et al. 2012). This has led to growing interest in selective estrogen receptor modulators (SERMs) as safer alternatives.

Raloxifene, a first‐generation SERM, exhibits tissue‐selective estrogen receptor modulation, acting as an agonist in the brain and bones, while serving as an antagonist in the breast and uterus, thus offering a favorable safety profile (Shang and Brown 2002; Moen and Keating 2008). Increasing evidence supports its potential for improving mood and psychotic symptoms in postmenopausal women (Wong et al. 2003), and emerging studies have investigated its efficacy in men and premenopausal women as well (Kulkarni et al. 2016; Khodaie‐Ardakani et al. 2015; Brand et al. 2023). However, the results of randomized controlled trials (RCTs) assessing raloxifene for schizophrenia have been inconsistent, particularly across different sexes and disease severities. Therefore, an updated and comprehensive meta‐analysis is needed to clarify its therapeutic efficacy and identify appropriate patient populations.

In 2018, de Boer et al. published a systematic review and meta‐analysis, which remains one of the most influential studies to date (de Boer et al. 2018). Their analysis included nine RCTs with a total of 561 patients diagnosed with schizophrenia spectrum disorders. The results demonstrated that raloxifene augmentation was significantly more effective than placebo in reducing overall symptom severity (Hedges’ *g* = 0.57, *p* = 0.009). Significant improvements were also observed in PANSS positive (*g* = 0.32, *p* = 0.02), negative (*g* = 0.40, *p* = 0.02), and general (*g* = 0.46, *p* = 0.01) symptom subscales. However, their study did not find a significant effect of raloxifene on comorbid depression or cognitive function. Despite certain limitations, the findings of de Boer et al. clearly highlight the potential of raloxifene as an adjunctive treatment for schizophrenia, thereby providing a valuable basis for further research into its efficacy across diverse patient subgroups.

Building on this background, the present study aims to update and expand upon existing literature by including more recent RCTs that specifically examine the effects of raloxifene on positive and negative symptoms of schizophrenia. The analysis focused on changes in symptom severity as measured by the PANSS. We also conducted subgroup analyses based on dosage and illness severity to provide a more comprehensive understanding of the clinical applications of raloxifene.

## 2 Material and Methods

### 2.1 Search Strategy

We comprehensively searched PubMed, Embase, and Cochrane databases for RCTs published until May 31, 2024. The criteria for the selected articles were based on the PICO principles (participants, interventions, comparisons, and outcomes). We used a combination of Medical Subject Headings and keywords (raloxifene) AND (schizophrenia). The detailed keyword list is provided in Box 1. Studies were not limited to those published in English. This study was prospectively registered in PROSPERO (registration number: CRD42024560237).

All identified articles were imported into EndNote software (version X8, Thomson Research Soft, CT, USA) to automatically remove duplicate records. Two reviewers (Tzu‐Rong Peng and Ming‐Chia Lee) independently screened all titles and abstracts to evaluate the relevance of each article. Furthermore, we searched the available bibliographies and review articles to identify additional potential articles for inclusion. Any disagreements were resolved by consensus and consultation with a third reviewer (Shih‐Ming Chen).

### 2.2 Inclusion and Exclusion Criteria

The inclusion criteria were as follows: (I) schizophrenia was diagnosed in accordance with the Diagnostic and Statistical Manual‐IV criteria; (II) patients were treated with any antipsychotic agent and also received a raloxifene or placebo treatment; and (III) the treatment effect was measured using the Positive and Negative Syndrome Scale (PANSS) or scale for assessment of negative symptoms (SANS) scores. The exclusion criteria were as follows: (I) the study was a review, survey, observational study, case series, or case report; and (II) there were no report‐related data. The selection criteria for the PICOS format were as follows:

- **Population**: Patients diagnosed with schizophrenia
- **Intervention**: Treatment with any antipsychotic agent and add‐on raloxifene
- **Comparison**: Placebo
- **Outcome**: Change in PANSS or SANS scores
- **Study design**: RCT

### 2.3 Methodological Quality Appraisal

Two authors (Tzu‐Rong Peng and Jian‐Ying Wang) independently assessed the risk of bias using the Cochrane risk of bias tool for randomized trials version 2.0 (Sterne et al. 2019). Bias assessment focuses on different aspects of trial design, conduct, and reporting. Within each area, a series of questions are designed to elicit information about trial characteristics relevant to the risk of bias. Based on the answers to the signal questions, the algorithm generates recommendations about the risk of bias in each area. The judgment can be “low” (green) or “high” (red) risk of bias, or it can express “some concerns” (yellow). A low risk of attrition bias was determined if the dropout rate was below 20%, owing to the limited number of participants in each study. To evaluate the reporting bias, the protocols of each study were reviewed on clinicaltrials.gov and biomedcentral.com. The third author (Shih‐Ming Chen) resolved any disagreements between the two authors. However, systematic reviewers conducting pairwise meta‐analyses sometimes encounter multi‐arm studies. To include these studies, two or more arms are often combined, or the control arm is split to avoid a unit‐of‐analysis error. If they must be counted together, “splitting the shared group” is used to avoid double counting the shared group (Rücker et al. 2017).

### 2.4 Data Extraction

Two researchers (Ming‐Chia Lee and Hung‐Hong Lin) independently extracted the data, and a third reviewer (Tzu‐Rong Peng) resolved disagreements. The following information was collected using a standardized data extraction form: authors, year of publication, diagnosis, diagnostic criteria, dose of raloxifene, duration of interventions, mean age, sex (male/female), antipsychotic agents, and outcome measures. Data on the demographic characteristics of the study participants were also extracted.

### 2.5 Statistical Analysis

This meta‐analysis was performed in accordance with the Preferred Reporting Items for Systematic Reviews and Meta‐Analyses (PRISMA) guidelines (Page et al. 2021). The PRISMA checklist is provided in Table S1. Statistical analyses were conducted using RevMan software (Cochrane Review Manager Version 5.4, Oxford, UK), Comprehensive Meta‐Analysis software version 3 (Biostat, Englewood, New Jersey, USA), and the Restricted Maximum Likelihood (REML) method for estimating between‐study variance to enhance the precision and robustness of the random‐effects model.

Continuous variables were summarized either as mean difference (MD) or standardized mean difference (SMD), both with 95% confidence intervals (CI). Specifically, the MD was used when outcomes were measured using the same scale across studies (e.g., PANSS total score), allowing direct comparison of MDs. In contrast, the SMD was applied to combine results from studies using different scales or versions of scales assessing similar constructs, by standardizing MDs across studies based on the pooled standard deviation. The pooled estimates of MD or SMD were initially calculated using the DerSimonian–Laird (DL) random‐effects model under the assumption of significant heterogeneity (DerSimonian and Laird 1986). However, recognizing the potential limitations of the DL method, particularly in meta‐analyses with a moderate number of studies, we also applied the Restricted Maximum Likelihood (REML) method to estimate the between‐study variance (*τ* ^2^) as part of a sensitivity analysis. REML is known to provide more accurate and less biased estimates of heterogeneity and better coverage of confidence intervals compared to the DL method. Both approaches are reported, with REML estimates provided in the sensitivity analysis.

Statistical heterogeneity among the included studies was assessed using Cochran's Q‐test, the *I* ^2^ statistic, and the between‐study variance Tau^2^ (*τ* ^2^). *I* ^2^ values were used to estimate the percentage of total variation across studies attributable to heterogeneity rather than chance, with values above 50% generally indicating moderate to high heterogeneity. Cochran's Q‐test was used to assess the presence of heterogeneity, with a *p* value < 0.10 considered statistically significant. Significant heterogeneity was defined as *I* ^2^ > 50% and/or Q‐test *p* value < 0.10. In addition, *τ* ^2^ was calculated to estimate the absolute variance of true effect sizes between studies under the random‐effects model. Although *τ* ^2^ is a scale‐dependent measure and lacks universally accepted interpretation thresholds, it was used to quantify between‐study variance in conjunction with *I* ^2^ and Cochran's Q statistics. The interpretation of *τ* ^2^ was contextualized based on the scale of the outcome measure (e.g., PANSS). All *p* values < 0.05 were considered statistically significant unless otherwise specified. Publication bias was assessed using funnel plots, Egger's regression test, and Begg's rank correlation test. Symmetrical funnel plots, along with *p* values greater than 0.05 from Egger's and Begg's tests, were interpreted as indicating no significant evidence of publication bias. Conversely, asymmetry in funnel plots or *p* values ≤ 0.05 would suggest possible publication bias. Subgroup outcome analyses were performed based on the dose and duration of raloxifene administration. Subgroup analysis also distinguished the effects of using raloxifene in women in menopause and other statuses. Sensitivity analyses included leave‐one‐out analysis and comparison of results obtained using different heterogeneity estimation methods (DL vs. REML).

## 3 Results

### 3.1 Study Selection and Study Characteristics

**FIGURE 1:**
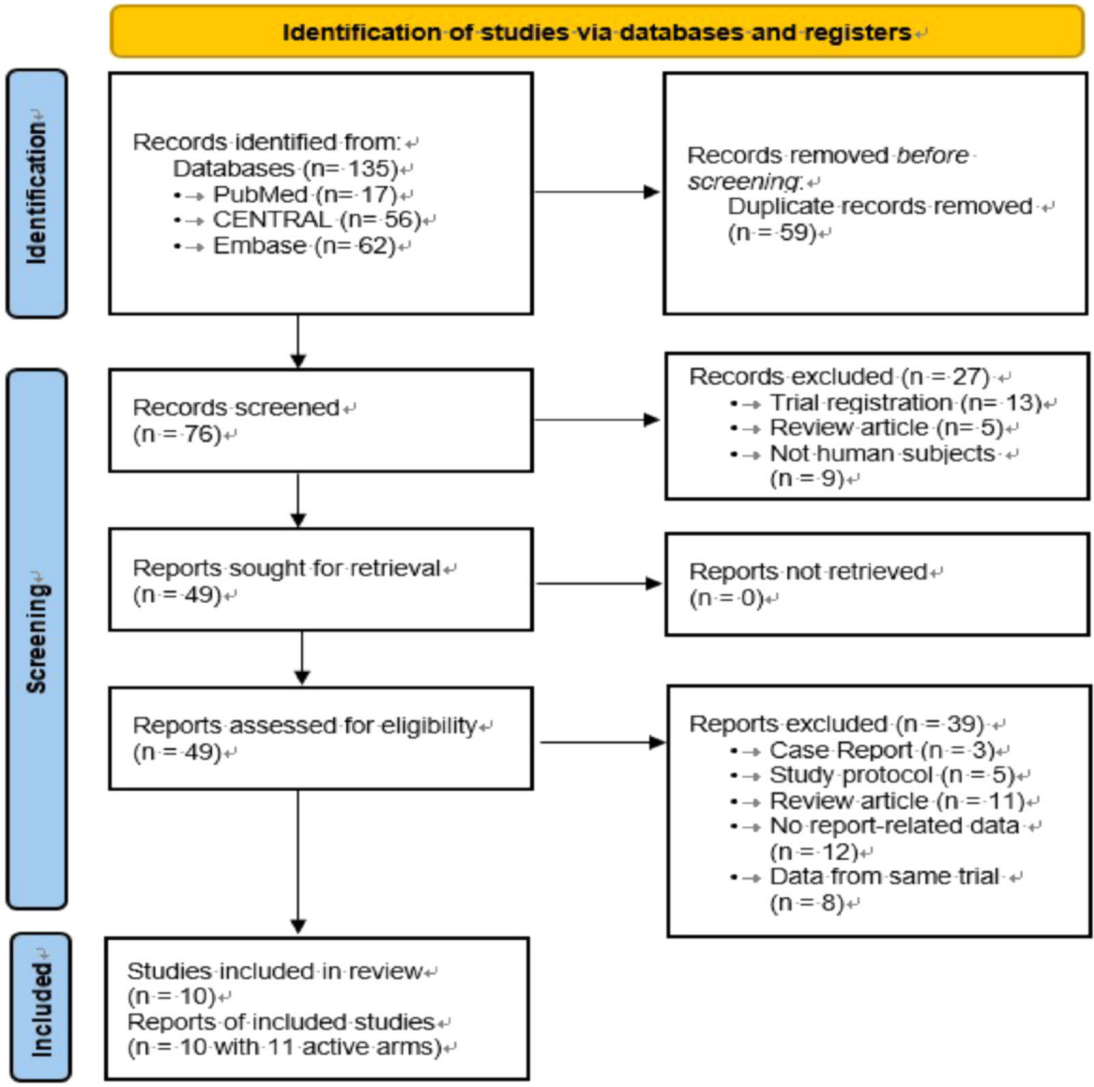
PRISMA flow diagram for study selection (Page et al. 2021). *Consider, if feasible to do so, reporting the number of records identified from each database or register searched (instead of the total number across all databases and/or registers). **If automation tools were used, indicate how many records were excluded by a human and how many were excluded by an automation tool.

The flow of the primary studies from electronic searches, screening for eligibility, and inclusion is shown in Figure 1. Among the 135 initially retrieved records, 59 were marked as duplicates by the automation tools, 27 were excluded after reviewing the title and abstract because their topics were unrelated to this research article, and 40 were excluded after reviewing the full texts of 49 articles. Specifically, 11 review articles, 12 studies without outcomes of interest, 8 studies with data from the same trial, 5 study protocols, and 3 case reports were excluded. Finally, ten studies were included in the meta‐analysis.

Table 1 summarizes the main characteristics of the included studies. This meta‐analysis included 675 patients with schizophrenia who were divided into raloxifene (*n* = 345) and control (*n* = 330) groups. The duration of the trials ranged from 6 to 24 weeks. The mean age of patients with schizophrenia ranged from 32.4 to 61.7 years; the female sex was predominant (75.9%, 512 patients). All the included articles used PANSS scores to measure treatment effectiveness.

**TABLE 1:** Characteristics of the included studies.

First author/year	Diagnosis	Diagnostic criteria	Duration	Treatment (Drugs, mg/day)	Number of patients	Mean age, years	Sex (M/F)	Antipsychotic	Outcome
Kulkarni et al. (2010)	SZ/SAD/SFD	DSM‐IV	12 weeks	Raloxifene 60 mg	9	52.6	F (post)	Risperidone	PANSS
				Raloxifene 120 mg	13				
				Placebo	13				
Usall et al. (2011)	SZ	DSM‐IV	12 weeks	Raloxifene 60 mg	16	61.4	F (post)	Risperidone	PANSS
				Placebo	17				
Kianimehr et al. (2014)	SZ	DSM‐IV	8 weeks	Raloxifene 120 mg	23	61.2	F (post)	Risperidone	PANSS
				Placebo	23				
Weickert et al. (2015)	SZ/SAD	DSM‐IV	6 weeks	Raloxifene 120 mg	40	37.4	M, F (pre/post)	Antipsychotics	PANSS
				Placebo	39				
Kulkarni et al. (2016)	SZ/SAD	DSM‐IV	12 weeks	Raloxifene 120 mg	26	53	F (per/post)	Risperidone	PANSS
				Placebo	30				
Usall et al. (2016)	SZ	DSM‐IV	24 weeks	Raloxifene 60 mg	38	61.7	F (post)	Chlorpromazine	PANSS
				Placebo	32				
Weiser et al. (2017)	SZ/SAD	DSM‐IV	16 weeks	Raloxifene 120 mg	100	52.6	F (post)	Antipsychotics	PANSS
				Placebo	100				
Vila et al. (2019)	SZ	DSM‐IV	24 weeks	Raloxifene 60 mg	7	56.8	F (post)	Chlorpromazine	PANSS
				Placebo	7				
Khodaie‐Ardakani et al. (2015)	SZ	DSM‐IV‐TR	8 weeks	Raloxifene 120 mg	21	32.4	F (post)	Risperidone	PANSS
				Placebo	21				
Brand et al. (2023)	SZ/SAD/SFD	DSM‐IV	12 weeks	Raloxifene 120 mg	52	42	M, F (pre/post)	Antipsychotics	PANSS
				Placebo	48				

Abbreviations: DSM‐IV, Diagnostic and Statistical Manual of Mental Disorders 4th edition; DSM‐IV‐TR, Diagnostic and Statistical Manual of Mental Disorders 4th edition (Text Revision); PANSS, Positive and Negative Syndrome Scale; per, perimenopausal; post, postmenopausal; SAD, schizoaffective disorder; SFD, schizophreniform disorder; SZ, schizophrenia.

### 3.2 Quality Assessment

**FIGURE 2:**
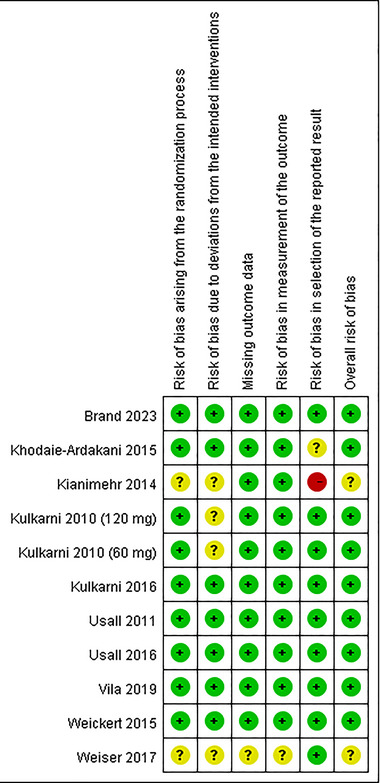
Risk‐of‐bias assessments for randomized clinical trials were included in the meta‐analysis.

Figure 2 shows the quality assessment results of the ten RCTs. Among them, six RCTs were found to have a low risk across all assessed domains. Notably, one study exhibited a concerning risk related to missing outcome data (Weiser et al. 2017), and one study had more than four domains categorized as having an unclear risk of bias (Weiser et al. 2017).

### 3.3 Efficacy

**FIGURE 3:**
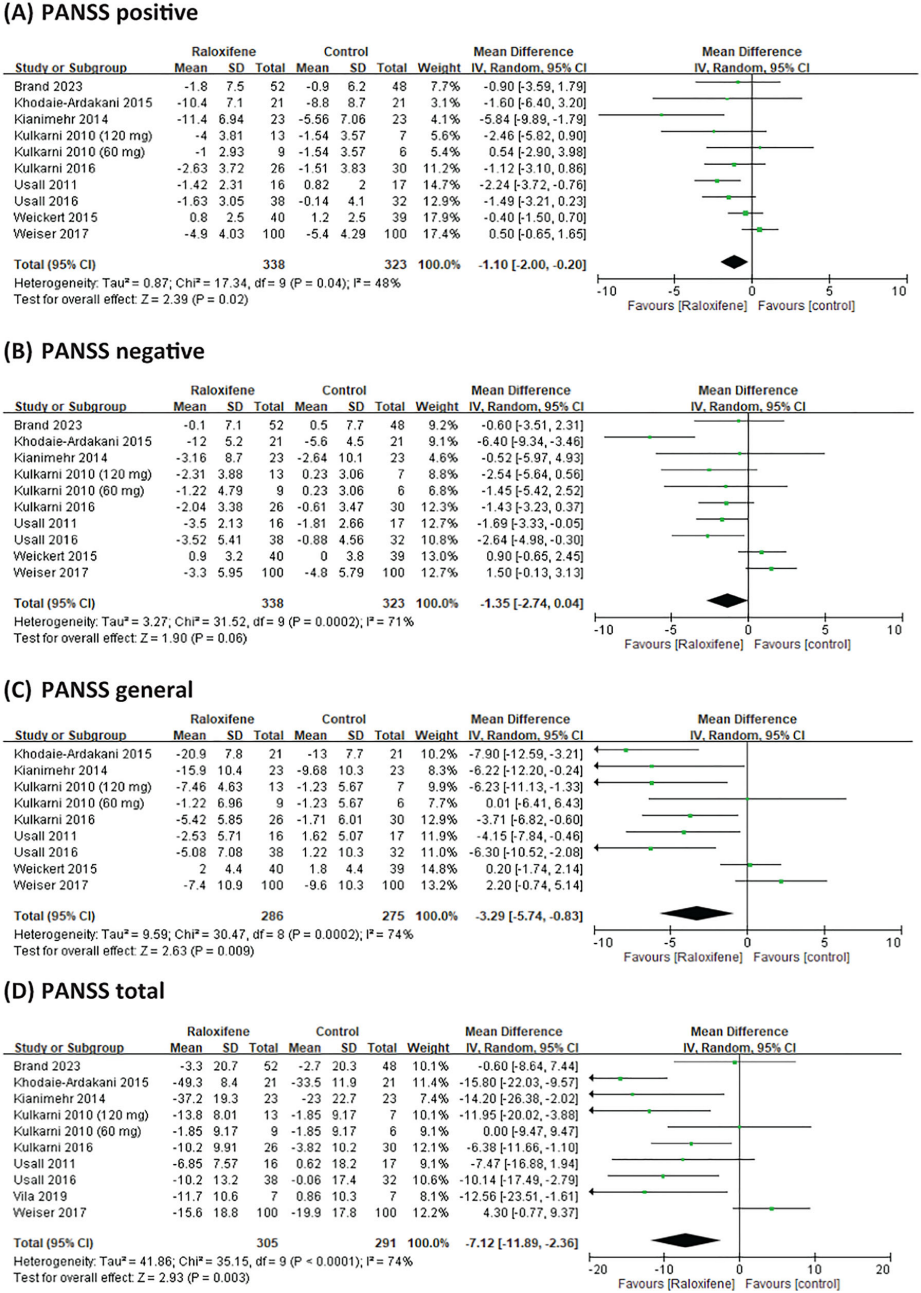
Forest plots for a between‐group meta‐analysis comparing psychiatric symptoms in patients with schizophrenia receiving or not receiving raloxifene as an adjunctive therapy.

Nine RCTs reported data on PANSS positive and negative scores; eight reported data on the PANSS general score, and nine studies reported data on the PANSS total score. Compared with the placebo group, raloxifene as an adjunctive therapy significantly improved the positive, general, and total PANSS scores, MD = −1.10 (95% CI = −2.00 to −0.20; *I* ^2^ = 48%; *p* = 0.02; *τ* ^2^ = 0.87), MD = −3.29 (95% CI = −5.74 to −0.83; *I* ^2^ = 74%; *p* = 0.009; *τ* ^2^ = 9.59), and MD = −7.12 (95% CI = −11.89 to −2.36; *I* ^2^ = 74%; *p* = 0.003; *τ* ^2^ = 41.86), respectively (Figure 3). For negative symptoms, the improvement showed a trend toward significance with an MD of −1.35 (95% CI: −2.74 to 0.04; *I* ^2^ = 71%; *p* = 0.06; *τ* ^2^ = 3.27) (Figure 3).

### 3.4 Subgroup Analysis

We conducted a subgroup analysis of different doses of raloxifene, duration, and menopausal status in women. In patients receiving adjuvant treatment for schizophrenia, the PANSS score showed no dose‐dependent raloxifene effects. However, the results showed that 60 mg of raloxifene was significantly different from 120 mg of raloxifene in improving the PANSS positive and negative scores (MD = −1.65, 95% CI = −2.79 to −0.51; MD = −1.95, 95% CI = −3.22 to −0.68) (Figure 4). The efficacy of raloxifene varied depending on the duration of the intervention. The PANSS‐positive, negative, and general scores in the raloxifene group were not significantly different from those in the placebo group at > 12 weeks (*p* > 0.05). However, at intervention durations of ≤ 12 weeks, the raloxifene group showed greater improvement in PANSS positive, negative, general and total scores than the control group (MD = −1.48, 95% CI = −2.41 to −0.39, *I* ^2^ = 34%; MD = −1.63, 95% CI = −3.15 to −0.12, *I* ^2^ = 66%; MD = −3.75, 95% CI = −6.34 to −1.16, *I* ^2^ = 67%; MD = −8.07, 95% CI = −12.7 to −3.44, *I* ^2^ = 58%;). In the subgroup analysis of postmenopausal women, the results showed that raloxifene significantly improved the PANSS general and total scores (MD = −3.31, 95% CI = −6.74 to −0.12, *I* ^2^ = 72%; MD = −6.84, 95% CI = −13.04 to −0.62, *I* ^2^ = 74%) (Table 2).

FIGURE 4Forest plots for a subgroup meta‐analysis comparing psychiatric symptoms in patients with schizophrenia receiving different doses of adjunctive raloxifene therapy.

**Figure:**
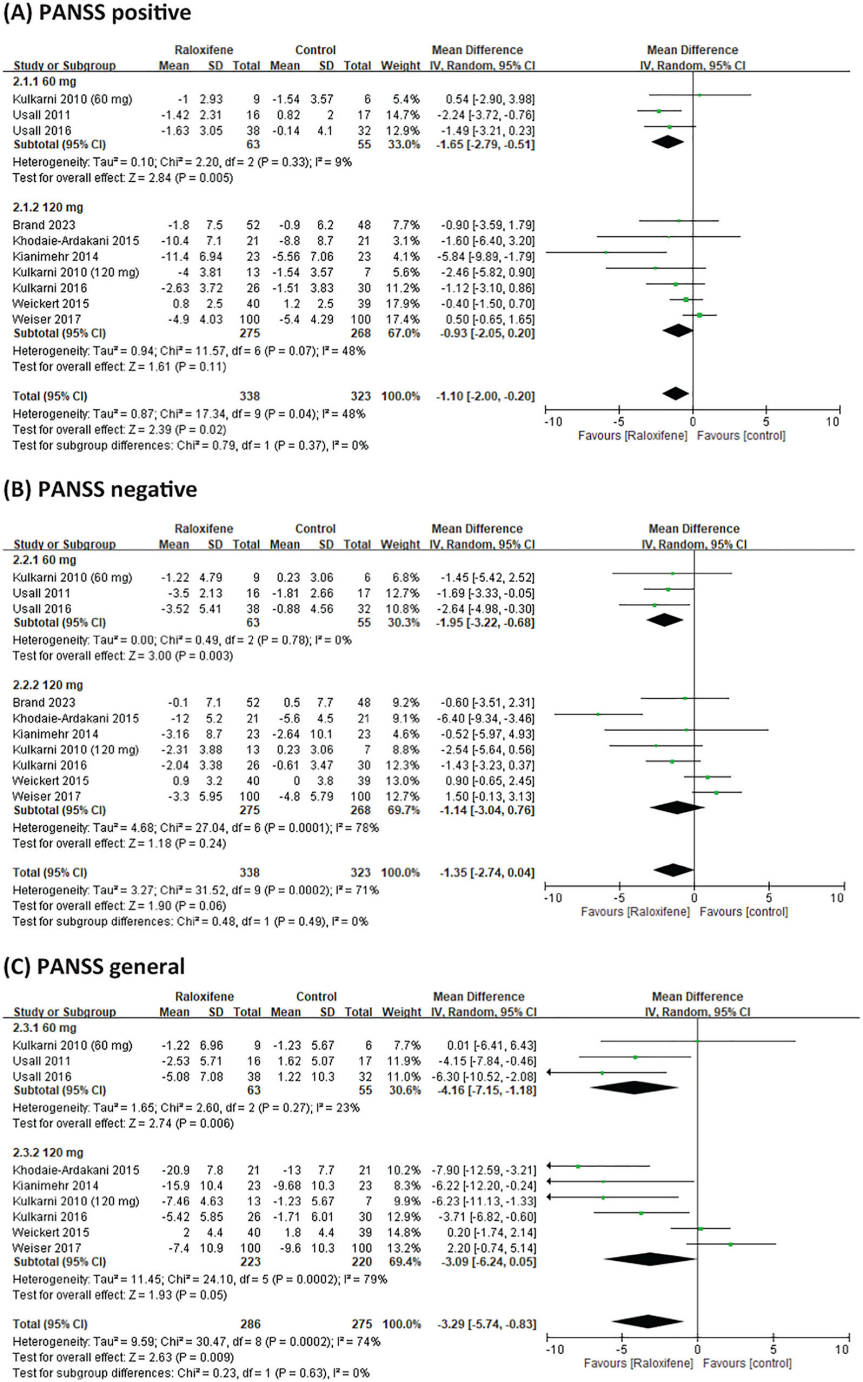

**Figure:**
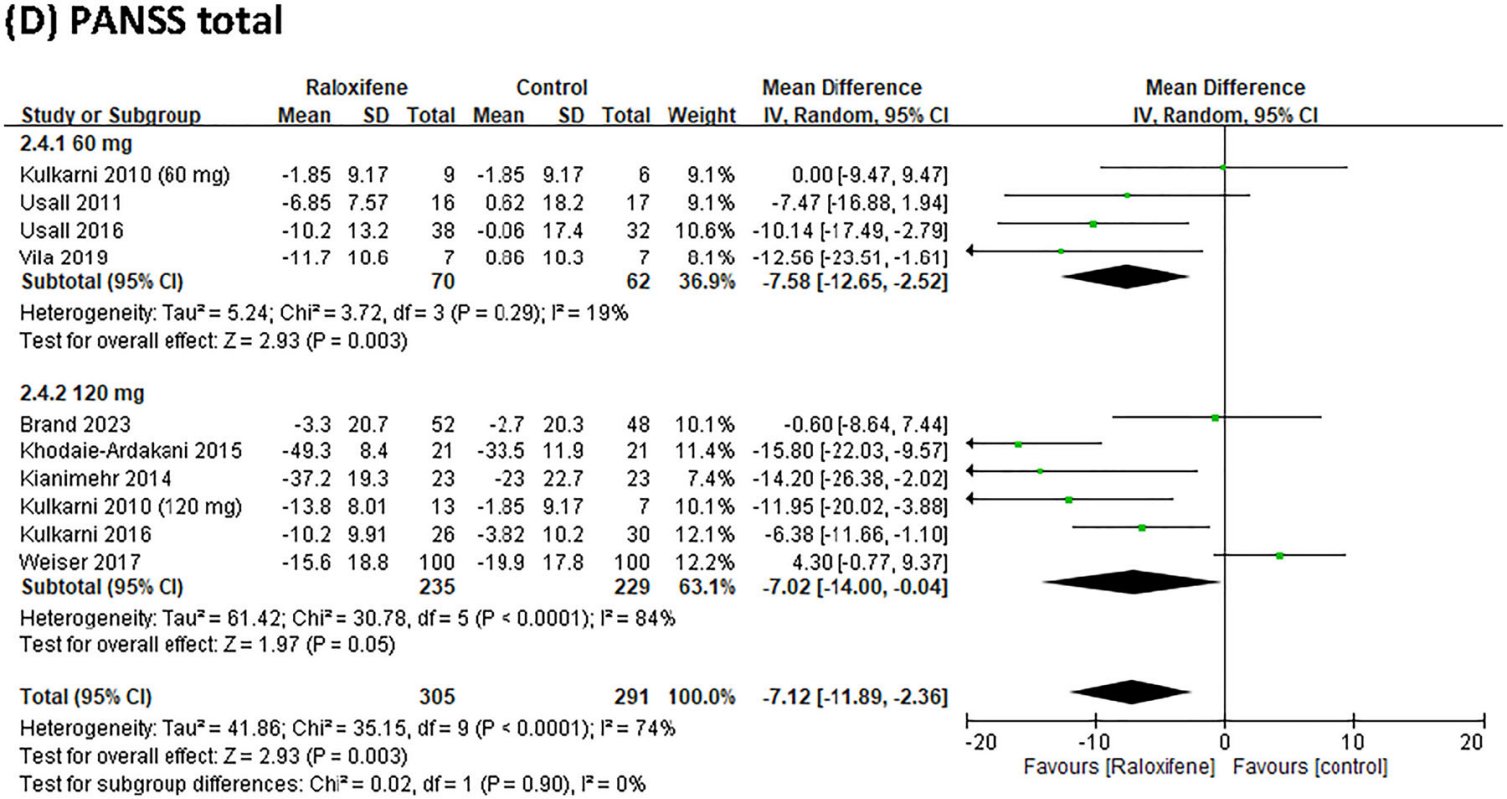

**TABLE 2:** Results of subgroup meta‐analysis of the treatment effect of adjunct raloxifene in patients with schizophrenia.

Outcomes	Number of studies	Summary MD (95% CI)	Results of the heterogeneity test		
			*I* ^2^	*p* value	τ2
PANSS positive					
Duration > 12	2	−0.39 (−2.33 to 1.55)	72%	0.69	1.42
Duration ≤ 12	7	−1.48 (−2.41 to −0.39)	34%	0.007	0.65
Postmenopausal women	5	−1.48 (−3.05 to 0.09)	70%	0.06	2.35
Mixed	4	−0.64 (−1.53 to 0.25)	0%	0.16	0.00
PANSS negative					
Duration > 12	2	−0.48 (−4.54 to 3.57)	88%	0.82	7.52
Duration ≤ 12	7	−1.63 (−3.15 to −0.12)	66%	0.03	2.82
Postmenopausal women	5	−1.13 (−2.79 to 0.53)	60%	0.18	2.32
Mixed	4	−1.72 (−4.45 to 1.02)	84%	0.22	6.40
PANSS general					
Duration > 12	2	−1.91 (−10.23 to 6.42)	90%	0.65	32.68
Duration ≤ 12	6	−3.75 (−6.34 to −1.16)	67%	0.005	7.48
Postmenopausal women	5	−3.31 (−6.74 to −0.12)	72%	0.06	12.76
Mixed	3	−3.40 (−7.78 to 0.99)	83%	0.13	12.20
PANSS total					
Duration > 12	3	−5.54 (−17.05 to 5.97)	86%	0.35	87.15
Duration ≤ 12	6	−8.07 (−12.7 to −3.44)	58%	0.0006	21.67
Postmenopausal women	6	−6.84 (−13.04 to −0.62)	74%	0.03	49.58
Mixed	3	−7.85 (−15.98 to 0.29)	79%	0.06	40.59

Abbreviations: CI, confidence interval; MD, mean differences.

### 3.5 Sensitivity Analysis

To assess the robustness of the pooled results and evaluate the influence of individual studies, a leave‐one‐out sensitivity analysis was conducted for the PANSS positive, negative, general, and total scores. This approach systematically excluded one study at a time to determine whether any single study had a disproportionate impact on the overall effect size. The results are presented in Table 3. For the PANSS positive and negative scores, the findings remained generally consistent with the overall pooled results. However, for the PANSS positive score, when the study by Usall et al. (2011) was excluded, the effect of add‐on raloxifene therapy became trend‐significant (MD = −0.86, 95% CI: −1.77 to 0.06; *I* ^2^ = 39%; *p* = 0.07). For the PANSS negative scores, stability was maintained with no significant differences when any single study was individually omitted, and the effect remained consistently non‐significant. In contrast, the pooled effects for PANSS general and total scores remained stable across all iterations, indicating that no single study substantially affected the overall estimates. Specifically, the MDs for PANSS general scores ranged from −2.72 to −4.07, and for PANSS total scores from −5.89 to −8.71, with overlapping confidence intervals and low variability across exclusions.

**TABLE 3:** The sensitivity analysis with (A) PANSS positive (B) PANSS negative (C) PANSS general and (D) PANSS total score (Leave One Out) for a between‐group meta‐analysis comparing psychiatric symptoms in patients with schizophrenia receiving or not receiving raloxifene as an adjunctive therapy.

(A) PANSS positive score						
Studies	MD	Lower CI	Upper CI	I^2^ (%)	*p* value	τ2
Overall	−1.10	−2.00	−0.20	48	0.02	0.87
Omitting						
Brand et al. (2023)	−1.14	−2.13	−0.16	54	0.02	1.04
Khodaie‐Ardakani et al. (2015)	−1.10	−2.05	−0.15	54	0.02	0.99
Kianimehr et al. (2014)	−0.84	−1.59	−0.08	29	0.03	0.36
Kulkarni et al. (2010)	−1.14	−2.13	−0.14	56	0.03	1.00
Kulkarni et al. (2016)	−1.13	−2.15	−0.11	54	0.03	1.09
Usall et al. (2011)	−0.86	−1.77	0.06	39	0.07	0.67
Usall et al. (2016)	−1.07	−2.09	−0.05	52	0.04	1.06
Weickert et al. (2015)	−1.30	−2.90	−0.20	52	0.02	1.26
Weiser et al. (2017)	−1.37	−2.21	−0.54	25	0.001	0.38

(B) PANSS negative score						
Studies	MD	Lower CI	Upper CI	I^2^ (%)	*p* value	τ2
Overall	−1.35	−2.74	0.04	71	0.06	3.27
Omitting						
Brand et al. (2023)	−1.45	−2.97	0.08	75	0.06	3.67
Khodaie‐Ardakani et al. (2015)	−0.77	−1.89	0.35	53	0.18	1.42
Kianimehr et al. (2014)	−1.40	−2.86	0.06	75	0.06	3.48
Kulkarni et al. (2010)	−1.24	−2.83	0.36	77	0.13	3.76
Kulkarni et al. (2016)	−1.37	−2.97	0.23	74	0.09	4.04
Usall et al. (2011)	−1.33	−2.94	0.27	74	0.10	4.04
Usall et al. (2016)	−1.20	−2.70	0.30	72	0.12	3.43
Weickert 2015	−1.69	−3.17	−0.20	68	0.03	3.21
Weiser et al. (2017)	−1.75	−3.11	−0.38	63	0.01	2.48

(C) PANSS general score						
Studies	MD	Lower CI	Upper CI	I^2^ (%)	*p* value	τ2
Overall	−3.29	−5.74	−0.83	74	0.009	9.59
Omitting						
Khodaie‐Ardakani et al. (2015)	−2.72	−5.14	−0.29	71	0.03	7.96
Kianimehr et al. (2014)	−3.02	−5.59	−0.45	75	0.02	9.70
Kulkarni et al. (2010)	−3.26	−6.04	−0.49	78	0.02	10.33
Kulkarni et al. (2016)	−3.27	−6.08	−0.46	76	0.02	11.54
Usall et al. (2011)	−3.20	−5.93	−0.47	76	0.02	10.86
Usall et al. (2016)	−2.90	−5.46	−0.34	73	0.03	9.15
Weickert 2015	−3.90	−6.59	−1.21	69	0.005	9.93
Weiser et al. (2017)	−4.07	−6.50	−1.65	67	0.001	7.50

(D) PANSS total score						
Studies	MD	Lower CI	Upper CI	I^2^ (%)	*p* value	τ2
Overall	−7.12	−11.89	−2.36	74	0.003	41.86
Omitting						
Brand et al. (2023)	−7.87	−13.03	−2.72	76	0.003	44.88
Khodaie‐Ardakani et al. (2015)	−5.89	−10.45	−1.33	67	0.01	30.88
Kianimehr et al. (2014)	−6.56	−11.55	−1.58	76	0.01	42.55
Kulkarni et al. (2010)	−7.37	−12.92	−1.81	78	0.009	47.54
Kulkarni et al. (2016)	−7.29	−12.89	−1.69	77	0.01	54.62
Usall et al. (2011)	−7.12	−12.33	−1.90	77	0.007	46.81
Usall et al. (2016)	−6.80	−12.05	−1.54	76	0.01	46.90
Vila et al. (2019)	−6.65	−11.70	−1.60	76	0.01	43.64
Weiser et al. (2017)	−8.71	−12.40	−5.02	47	0.0001	14.23

Abbreviations: CI, confidence interval; MD, mean difference.

In addition, to explore alternative approaches for estimating between‐study variance, we conducted a sensitivity analysis using the REML method. REML is recognized for producing more accurate and less biased estimates of heterogeneity, particularly in meta‐analyses with a moderate number of studies. The REML‐based random‐effects models generated pooled estimates that were highly consistent with those derived from the DL method in both magnitude and direction of effect. This concordance further reinforces the robustness and reliability of our findings, suggesting that our results are not sensitive to the choice of a between‐study variance estimation method. Detailed REML‐based estimates are presented in Figures S1–S4.

### 3.6 Raloxifene Safety in Schizophrenia Treatment

Nine studies reported adverse effects of schizophrenia treatment, and only one study did not report any adverse effects (Vila et al. 2019). The reported adverse effects in the add‐on raloxifene and control groups were sweating, weight gain, headache, sex dysfunction, tremors, restless leg syndrome, dry mouth, loss of appetite, drowsiness, fatigue, constipation, and insomnia. However, the differences between the two groups were not statistically significant. None of the included trials reported any serious adverse events.

### 3.7 Publication Bias

**FIGURE 5:**
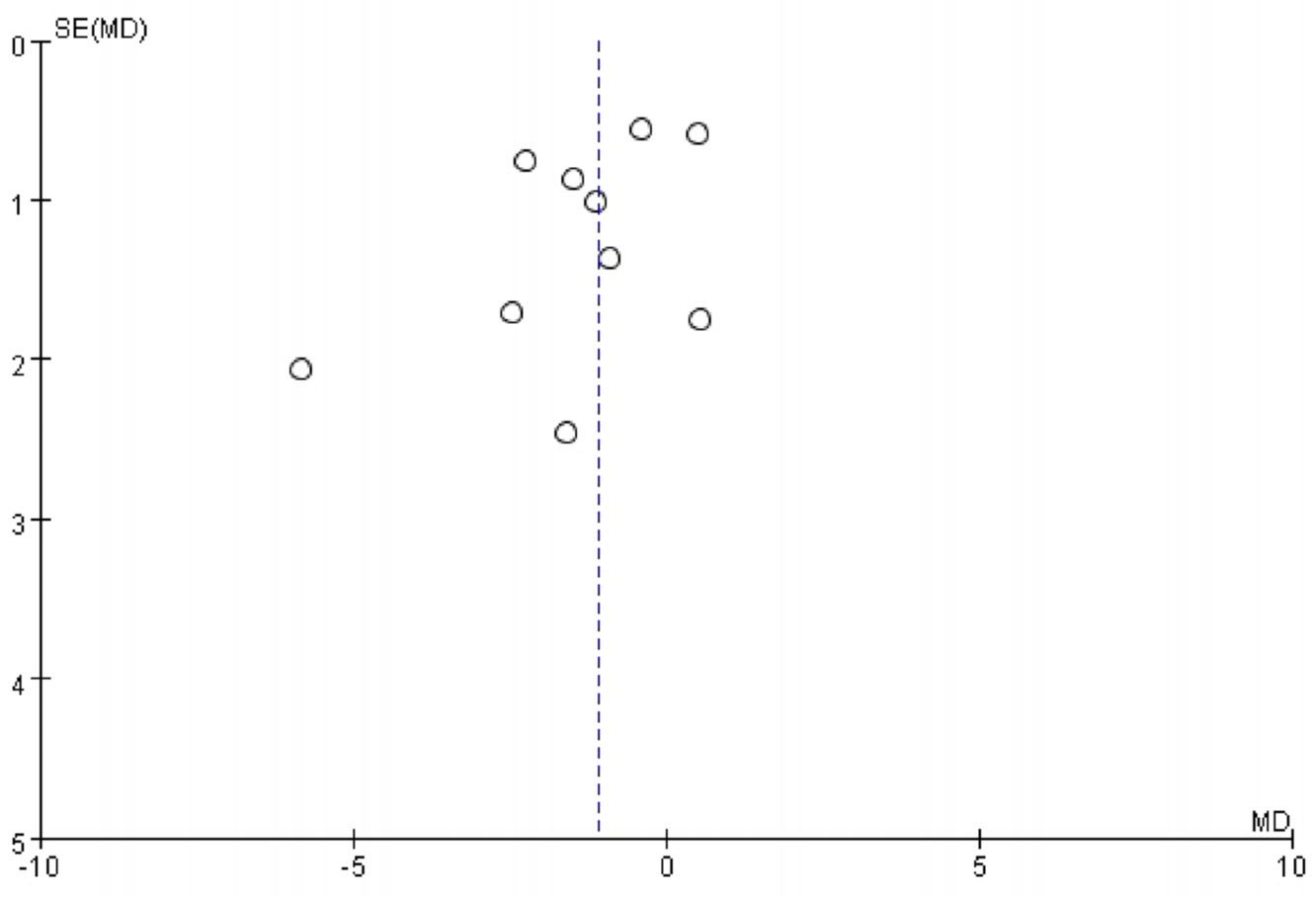
Funnel plot of potential publication bias (PANSS Positive).

Publication bias was evaluated by visually inspecting funnel plots of effect sizes against their standard errors for each included trial (Figure 5). In addition, Egger's regression test and Begg's rank correlation test were conducted to statistically assess potential bias. Both tests yielded non‐significant results (*p* > 0.05), indicating no evidence of significant publication bias in the included studies.

## 4 Discussion

This meta‐analysis systematically updates the literature on the efficacy of raloxifene as an adjunctive antipsychotic treatment for schizophrenia. Consistent with the findings of de Boer et al. (2018), our analysis revealed that adjuvant raloxifene significantly improves total symptom severity, as well as positive, negative, and general symptom domains as measured by PANSS scores. Notably, while previous meta‐analyses (Li et al. 2023; Wang et al. 2018) primarily focused on postmenopausal women, our study broadens the evidence base by including male and premenopausal female participants (Khodaie‐Ardakani et al. 2015; Brand et al. 2023), thereby providing a more comprehensive, population‐based perspective on the clinical benefits of raloxifene. Although the majority of patients in our included studies were still postmenopausal women (80.9%), our inclusion criteria allow for a more generalized understanding of its potential efficacy across sex and menopausal status. Raloxifene, a SERM, is hypothesized to exert therapeutic effects in schizophrenia primarily through modulation of the dopaminergic system. The mechanism of action of raloxifene in schizophrenia can be explained by its ability to change dopamine‐related behaviors associated with the disorder, which has primarily been demonstrated in healthy male or ovariectomized female rodents  (Sbisa et al. 2018). Preclinical animal studies support this mechanism and demonstrate that raloxifene's effects on dopamine‐related behaviors vary depending on sex, neurodevelopmental background, and physiological context. For example, Purves‐Tyson et al. (2015) reported that raloxifene potentiated amphetamine‐induced hyperlocomotion in healthy adult male rats—a behavior commonly used to model psychosis‐like dopaminergic hyperactivity. This finding suggests that raloxifene may enhance sensitivity to dopaminergic stimulation under normal physiological conditions.

In contrast, Debs et al. (2024) showed that raloxifene reduced amphetamine‐induced hyperlocomotion in both male and female offspring exposed to maternal immune activation (a neurodevelopmental model of dopamine dysregulation), highlighting raloxifene's context‐dependent effects: it may either enhance or attenuate dopaminergic activity depending on the underlying neurobiological state. Evidence of dopaminergic modulation comes from Gogos and van den Buuse (2015), who found that raloxifene prevented prepulse inhibition (PPI) deficits—an indicator of impaired sensorimotor gating often observed in schizophrenia—induced by the D1/D2 agonist apomorphine in ovariectomized rats. Notably, high‐dose raloxifene reduced apomorphine‐induced PPI deficits to only 4%, suggesting that raloxifene can mitigate dopamine‐triggered disruptions in sensorimotor processing. These findings collectively support the idea that raloxifene acts via dopaminergic pathway modulation, potentially through receptor‐level or downstream signaling effects. While 17β‐estradiol is known to affect dopamine synthesis and release, raloxifene may exert its effects primarily at the postsynaptic level, as proposed by Gogos et al. (2024). This mechanistic pathway could underlie its therapeutic potential in schizophrenia. SERMs do not carry the side effects of estrogen because they agonize estrogen receptors in the brain and bones but not in the sex organs. Therefore, SERMs such as raloxifene may have therapeutic benefits in patients of both sexes with schizophrenia without being harmful to gynecological tissues or having feminizing effects (de Boer et al. 2018). Therefore, raloxifene can be used as an auxiliary treatment for schizophrenia regardless of sex.

Our study found that 60 mg of daily raloxifene significantly improved PANSS positive, negative, general, and total scores. However, the 120 mg daily raloxifene group showed significant improvements in PANSS general and total scores. The possible reason is that the studies included patients with mild‐to‐moderate schizophrenia who all used raloxifene 60 mg (Kulkarni et al. 2010; Usall et al. 2011; Usall et al. 2016; Vila et al. 2019). However, most studies using 120 mg of raloxifene included patients with severe schizophrenia (Khodaie‐Ardakani et al. 2015; Kianimehr et al. 2014; Weiser et al. 2017). Therefore, our study found that raloxifene 120 mg was not effective in improving PANSS in patients with severe schizophrenia. Raloxifene may be used in patients with mild‐to‐moderate schizophrenia; however, it is not currently recommended for patients with severe schizophrenia, even at high doses (120 mg).

In this study, we found significant heterogeneity in the pooled analysis of PANSS scores (*I* ^2^ = 48%–74%); therefore, we performed a sensitivity analysis. After excluding one outlier study (Weiser et al. 2017), heterogeneity was reduced (*I* ^2^ = 25%–67%), and the integrated analysis results became more robust. In the study by Weiser et al. (2017), which included patients diagnosed with severe schizophrenia, the results did not support the use of raloxifene to reduce symptoms in women with severe decompensated postmenopausal schizophrenia. These results suggest that raloxifene may effectively treat patients with mild symptoms. However, the same problem was observed for the duration of raloxifene treatment. Only two or three studies on raloxifene treatment for more than 12 weeks exist, one of which is Weser's study (Usall et al. 2016). In addition to the high heterogeneity of the results and the small number of included articles. Therefore, the conclusion that the raloxifene treatment duration should be more than 12 weeks requires further investigation. In addition, both our study and that of de Boer et al. observed moderate to high heterogeneity in the PANSS efficacy analyses. However, our further sensitivity analysis offered a new perspective: excluding the study by Weiser et al. (2017) significantly reduced some heterogeneity (for example, *I* ^2^ for PANSS positive score decreased from 48% to 25%). The study by Weiser et al. (2017) included patients with more severe illness, strongly suggesting that disease severity may be an important source of heterogeneity and supporting the inference that raloxifene may be more effective in patients with mild to moderate schizophrenia. While de Boer et al. also assessed heterogeneity and examined outliers, their reported literature did not include a similar sensitivity analysis or draw explicit conclusions regarding disease severity as a heterogeneity factor.

Antipsychotic medications are generally effective in alleviating the positive symptoms of schizophrenia; however, negative and cognitive symptoms often persist, substantially impairing patients' quality of life and functional outcomes. Schizophrenia is associated with a range of neurobiological abnormalities, including reduced hippocampal volume, decreased oligodendrocyte density, impaired synaptic connectivity, and dendritic atrophy of pyramidal neurons, as demonstrated in neuropathological and neuroimaging studies (Harrison 1999). A prior meta‐analysis demonstrated that raloxifene can improve schizophrenia symptoms, particularly in postmenopausal women (Wang et al. 2018). However, more recent clinical studies have yielded inconsistent results regarding its efficacy across broader schizophrenia‐spectrum populations, suggesting possible sex‐specific or hormone‐dependent effects (Wang et al. 2018; Brand et al. 2023).

In our analysis, we found that low‐dose, short‐term adjunctive raloxifene may confer therapeutic benefits, particularly for positive and negative symptoms. In contrast, higher doses or prolonged treatment may activate compensatory mechanisms or lead to reduced efficacy, potentially due to hormonal feedback regulation or receptor desensitization. These observations highlight the need to further elucidate optimal dosing strategies and treatment duration, as well as to investigate sex‐specific neuroendocrine mechanisms underlying the effects of raloxifene. Compared to the widely cited meta‐analysis by de Boer et al. (2018), our study presents several key advantages. First, it incorporates the most recent RCTs published up to May 2024, thereby offering a more updated synthesis of the evidence. Second, our analysis includes more comprehensive and clinically informative subgroup analyses, particularly in relation to raloxifene dosage, treatment duration, and menopausal status. Notably, we found that a 60 mg daily dose significantly improved both positive and negative symptoms, while 120 mg did not demonstrate similar efficacy. This finding may reflect differences in illness severity among patients, as higher doses were more frequently used in trials involving individuals with severe schizophrenia. Third, our sensitivity analyses identified illness severity—specifically the inclusion of severely ill participants in the Weiser et al. (2017) study (Weiser et al. 2017)—as a major contributor to heterogeneity, suggesting that raloxifene may be more effective in patients with mild‐to‐moderate illness. This hypothesis was not explicitly explored in the previous meta‐analysis. Finally, we provide a more robust summary of safety outcomes by confirming that no serious adverse events were reported in any of the included trials. Collectively, these methodological enhancements not only strengthen the validity of our findings but also offer more precise clinical guidance for tailoring raloxifene use in patients with schizophrenia. Overall, our updated evidence supports and extends the conclusions of de Boer et al., reaffirming that raloxifene, when used as adjunctive therapy, can improve positive, negative, and general symptoms of schizophrenia. Furthermore, our exploration of heterogeneity—particularly through sensitivity analyses—offers important insights for future research, such as examining differential efficacy across varying illness severities or patient subgroups.

This meta‐analysis focused on the efficacy of adjunctive raloxifene in improving psychotic symptoms—specifically total, positive, negative, and general symptoms as measured by PANSS scores—cognitive symptoms were not within the scope of our quantitative analysis. Nonetheless, previous RCTs have investigated the effects of raloxifene on cognitive function in patients with schizophrenia, with some focusing specifically on postmenopausal women. Notably, Kulkarni et al. (2016) and Weiser et al. (2017) employed validated neurocognitive batteries such as the Repeatable Battery for the Assessment of Neuropsychological Status (RBANS) and the Brief Assessment of Cognition in Schizophrenia (BACS), respectively. Although these studies did not demonstrate statistically significant improvements in global cognitive scores, other trials—such as those by Weickert et al. (2015) and Brand et al. (2023)—reported domain‐specific and sex‐dependent effects. Improvements were observed in working memory and attention/processing speed, particularly among female participants. Conversely, Brand et al. (2023) found that raloxifene may exert a negative effect on verbal working memory in male patients by Week 38. These findings underscore the heterogeneous and potentially sex‐specific cognitive effects of raloxifene, which are important to consider in future trials. Future research should incorporate standardized cognitive outcome measures and stratified analyses by sex and disease characteristics to clarify the cognitive impact of raloxifene.

**Box 1**

PubMed

#1. (“raloxifen”[All Fields] OR “raloxifene hydrochloride”[MeSH Terms] OR (“raloxifene”[All Fields] AND “hydrochloride”[All Fields]) OR “raloxifene hydrochloride”[All Fields] OR “raloxifene”[All Fields] OR “raloxifene s”[All Fields]) AND (“schizophrenia”[MeSH Terms] OR “schizophrenia”[All Fields] OR

“schizophrenias”[All Fields] OR “schizophrenia s”[All Fields])

= 62

#2. #1 AND Filters: Randomized Controlled Trial

= 17

Embase

#1. ('raloxifene'/exp OR raloxifene) AND ('schizophrenia'/exp OR schizophrenia)

= 185

#2. #1 AND 'article'/it = 62

CENTRAL

#1. Raloxifene AND Schizophrenia

= 58

#2. Filters: Trial = 56

This study has some limitations that should be addressed. (1) The number of samples was relatively small. (2) Subgroup and sensitivity analyses were performed to reduce study inconsistencies because some of our analyses identified heterogeneity. Therefore, the results of this study should be interpreted with caution. (3) The duration of raloxifene treatment in all the included studies was relatively short (6–24 weeks); therefore, it was not possible to determine the long‐term effects and safety of raloxifene. (4) Most studies included postmenopausal women, and only a few included men and premenopausal women. Therefore, further studies are needed to confirm these results in men and premenopausal women.

## 5 Conclusion

This updated meta‐analysis demonstrates that adjunctive raloxifene is an effective strategy for improving positive, general, and total symptoms in patients with schizophrenia, particularly those with mild‐to‐moderate illness. A daily dose of 60 mg for at least 12 weeks showed the most consistent benefits, especially in postmenopausal women. Preliminary evidence also supports potential efficacy in men and premenopausal women, though further research is needed. Sensitivity analyses suggest that illness severity may influence treatment response, underscoring the importance of patient‐specific treatment strategies. Future RCTs with broader populations and longer follow‐ups are warranted to confirm these findings and guide clinical use.

## Author Contributions

T.R.P. and M.C.L. wrote the first draft of the manuscript. M.C.L. and H.H.L. searched databases and extracted the data. J.Y.W. and T.R.P. evaluated the risk of bias. T.R.P. and H.H.L. performed the statistical analysis. S.M.C. and M.C.L. critically revised the manuscript. All authors contributed to the final version of the manuscript.

## Conflicts of Interest

The authors declare no conflicts of interest.

## Peer Review

The peer review history for this article is available at https://publons.com/publon/10.1002/brb3.70649

## Supporting information




**Supplementary Figures**: brb370649‐sup‐0001‐SuppMat.docx

## Data Availability

All data, models, and code generated or used during the study appear in the submitted article.
