# Supplementary material for: Raloxifene as an Adjuvant Therapy for Patients With Schizophrenia: An Up‐To‐Date Systematic Review and Meta‐Analysis
Source: Brain Behav. 2025 Jul 20;15(7):e70649. doi: 10.1002/brb3.70649 (PMC12277536; doi:10.1002/brb3.70649)
Supplement: Supplementary file 1 — Supplementary Figures: brb370649‐sup‐0001‐SuppMat.docx [file BRB3-15-e70649-s001.docx]

| **PRISMA 2020 Checklist** | | | |
| --- | --- | --- | --- |
| **Section and Topic** | **Item #** | **Checklist item** | **Location where item is reported** |
| **TITLE** | | |  |
| Title | 1 | Identify the report as a systematic review. | Page 1 |
| **ABSTRACT** | | |  |
| Abstract | 2 | See the PRISMA 2020 for Abstracts checklist. | Page 1-2 |
| **INTRODUCTION** | | |  |
| Rationale | 3 | Describe the rationale for the review in the context of existing knowledge. | Page 3-4 |
| Objectives | 4 | Provide an explicit statement of the objective(s) or question(s) the review addresses. | Page 4-5 |
| **METHODS** | | |  |
| Eligibility criteria | 5 | Specify the inclusion and exclusion criteria for the review and how studies were grouped for the syntheses. | Page 6 |
| Information sources | 6 | Specify all databases, registers, websites, organisations, reference lists and other sources searched or consulted to identify studies. Specify the date when each source was last searched or consulted. | Page 6 |
| Search strategy | 7 | Present the full search strategies for all databases, registers and websites, including any filters and limits used. | Page 6 and Box 1 |
| Selection process | 8 | Specify the methods used to decide whether a study met the inclusion criteria of the review, including how many reviewers screened each record and each report retrieved, whether they worked independently, and if applicable, details of automation tools used in the process. | Figure 1, Page 6-7 |
| Data collection process | 9 | Specify the methods used to collect data from reports, including how many reviewers collected data from each report, whether they worked independently, any processes for obtaining or confirming data from study investigators, and if applicable, details of automation tools used in the process. | Page 8 |
| Data items | 10a | List and define all outcomes for which data were sought. Specify whether all results that were compatible with each outcome domain in each study were sought (e.g. for all measures, time points, analyses), and if not, the methods used to decide which results to collect. | Page 8 |
|  | 10b | List and define all other variables for which data were sought (e.g. participant and intervention characteristics, funding sources). Describe any assumptions made about any missing or unclear information. | Page 8 |
| Study risk of bias assessment | 11 | Specify the methods used to assess risk of bias in the included studies, including details of the tool(s) used, how many reviewers assessed each study and whether they worked independently, and if applicable, details of automation tools used in the process. | Page 8 |
| Effect measures | 12 | Specify for each outcome the effect measure(s) (e.g. risk ratio, mean difference) used in the synthesis or presentation of results. | Page 9 |
| Synthesis methods | 13a | Describe the processes used to decide which studies were eligible for each synthesis (e.g. tabulating the study intervention characteristics and comparing against the planned groups for each synthesis (item #5)). | Page 9-10 |
|  | 13b | Describe any methods required to prepare the data for presentation or synthesis, such as handling of missing summary statistics, or data conversions. | Page 9-10 |
|  | 13c | Describe any methods used to tabulate or visually display results of individual studies and syntheses. | Page 9-10 |
|  | 13d | Describe any methods used to synthesize results and provide a rationale for the choice(s). If meta-analysis was performed, describe the model(s), method(s) to identify the presence and extent of statistical heterogeneity, and software package(s) used. | Page 9-109 |
|  | 13e | Describe any methods used to explore possible causes of heterogeneity among study results (e.g. subgroup analysis, meta-regression). | Page 9-10 |
|  | 13f | Describe any sensitivity analyses conducted to assess robustness of the synthesized results. | Page 9-10 |
| Reporting bias assessment | 14 | Describe any methods used to assess risk of bias due to missing results in a synthesis (arising from reporting biases). | Page 7 |
| Certainty assessment | 15 | Describe any methods used to assess certainty (or confidence) in the body of evidence for an outcome. | Page 9 |
| **RESULTS** | | |  |
| Study selection | 16a | Describe the results of the search and selection process, from the number of records identified in the search to the number of studies included in the review, ideally using a flow diagram. | Page 11, figure 1 |
|  | 16b | Cite studies that might appear to meet the inclusion criteria, but which were excluded, and explain why they were excluded. | Figure 1 |
| Study characteristics | 17 | Cite each included study and present its characteristics. | Page 11  and table 1 |
| Risk of bias in studies | 18 | Present assessments of risk of bias for each included study. | Page 11-12 and figure 2 |
| Results of individual studies | 19 | For all outcomes, present, for each study: (a) summary statistics for each group (where appropriate) and (b) an effect estimate and its precision (e.g. confidence/credible interval), ideally using structured tables or plots. | Page 12-13 and table 1 |
| Results of syntheses | 20a | For each synthesis, briefly summarise the characteristics and risk of bias among contributing studies. | Page 12-14 and table 2 |
|  | 20b | Present results of all statistical syntheses conducted. If meta-analysis was done, present for each the summary estimate and its precision (e.g. confidence/credible interval) and measures of statistical heterogeneity. If comparing groups, describe the direction of the effect. | Page 12-14, figure 2 and 3 |
|  | 20c | Present results of all investigations of possible causes of heterogeneity among study results. | Page 12-14, figure 2 and 3 |
|  | 20d | Present results of all sensitivity analyses conducted to assess the robustness of the synthesized results. | Page 12-14, figure 3 and 4 |
| Reporting biases | 21 | Present assessments of risk of bias due to missing results (arising from reporting biases) for each synthesis assessed. | Page 12-14 and figure 5 |
| Certainty of evidence | 22 | Present assessments of certainty (or confidence) in the body of evidence for each outcome assessed. | Page 14 |
| **DISCUSSION** | | |  |
| Discussion | 23a | Provide a general interpretation of the results in the context of other evidence. | Page 15-16 |
|  | 23b | Discuss any limitations of the evidence included in the review. | Page 15-22 |
|  | 23c | Discuss any limitations of the review processes used. | Page 23 |
|  | 23d | Discuss implications of the results for practice, policy, and future research. | Page 23 |
| **OTHER INFORMATION** | | |  |
| Registration and protocol | 24a | Provide registration information for the review, including register name and registration number, or state that the review was not registered. | Page 6 |
|  | 24b | Indicate where the review protocol can be accessed, or state that a protocol was not prepared. | Page 6 |
|  | 24c | Describe and explain any amendments to information provided at registration or in the protocol. | Page 6 |
| Support | 25 | Describe sources of financial or non-financial support for the review, and the role of the funders or sponsors in the review. | Page 25 |
| Competing interests | 26 | Declare any competing interests of review authors. | Page 25 |
| Availability of data, code and other materials | 27 | Report which of the following are publicly available and where they can be found: template data collection forms; data extracted from included studies; data used for all analyses; analytic code; any other materials used in the review. | Page 25 |

*From:*  Page MJ, McKenzie JE, Bossuyt PM, Boutron I, Hoffmann TC, Mulrow CD, et al. The PRISMA 2020 statement: an updated guideline for reporting systematic reviews. BMJ 2021;372:n71. doi: 10.1136/bmj.n71

For more information, visit: <http://www.prisma-statement.org/>

**Table S1.** The PRISMA checklist


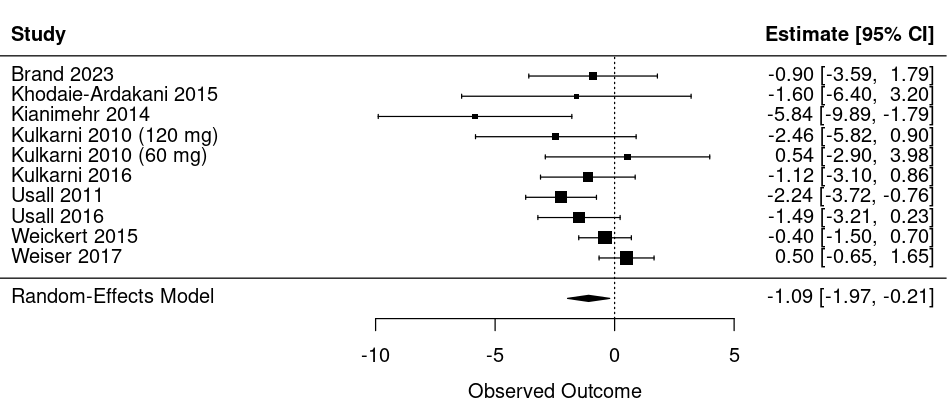


Random-Effects Model (k = 10; tau^2 estimator: REML)

logLik deviance AIC BIC AICc

-17.0184 34.0367 38.0367 38.4312 40.0367

tau^2 (estimated amount of total heterogeneity): 0.7958 (SE = 0.8713)

tau (square root of estimated tau^2 value): 0.8921

I^2 (total heterogeneity / total variability): 45.88%

H^2 (total variability / sampling variability): 1.85

Test for Heterogeneity:

Q(df = 9) = 17.3513, p-val = 0.0435

Model Results:

estimate se zval pval ci.lb ci.ub

-1.0872 0.4498 -2.4170 0.0157 -1.9689 -0.2056 *

---

Signif. codes: 0 ‘***’ 0.001 ‘**’ 0.01 ‘*’ 0.05 ‘.’ 0.1 ‘ ’ 1

**Figure S1.** Forest plot of a between-group meta-analysis comparing PANSS positive symptoms in schizophrenia patients receiving adjunctive raloxifene versus control, using a random-effects model with REML estimation.


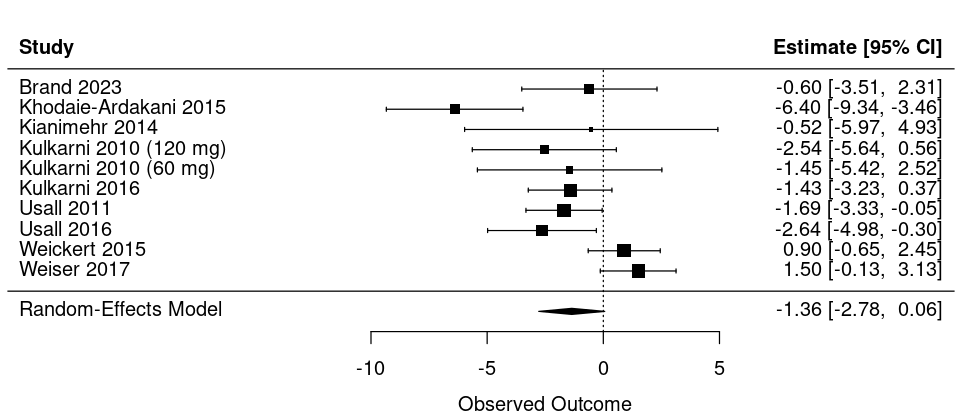


Random-Effects Model (k = 10; tau^2 estimator: REML)

logLik deviance AIC BIC AICc

-20.1580 40.3159 44.3159 44.7104 46.3159

tau^2 (estimated amount of total heterogeneity): 3.4515 (SE = 2.4141)

tau (square root of estimated tau^2 value): 1.8578

I^2 (total heterogeneity / total variability): 72.52%

H^2 (total variability / sampling variability): 3.64

Test for Heterogeneity:

Q(df = 9) = 31.4977, p-val = 0.0002

Model Results:

estimate se zval pval ci.lb ci.ub

-1.3591 0.7241 -1.8770 0.0605 -2.7782 0.0601 .

---

Signif. codes: 0 ‘***’ 0.001 ‘**’ 0.01 ‘*’ 0.05 ‘.’ 0.1 ‘ ’ 1

**Figure S2.** Forest plot of a between-group meta-analysis comparing PANSS negative symptoms in schizophrenia patients receiving adjunctive raloxifene versus control, using a random-effects model with REML estimation.


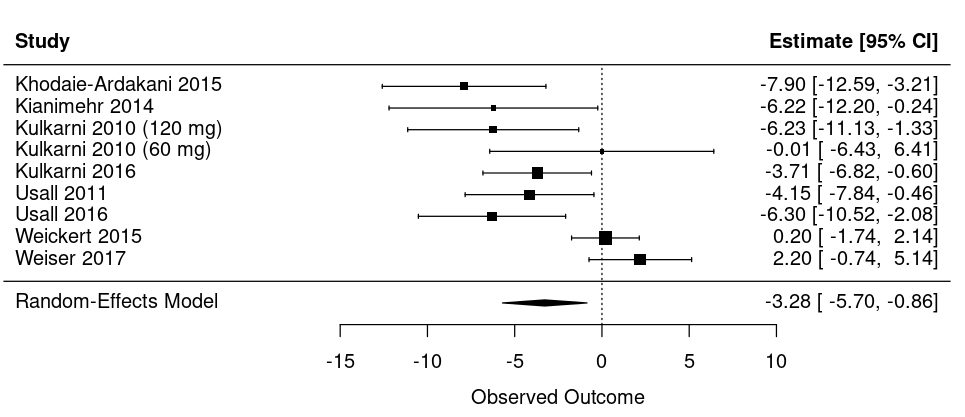


Random-Effects Model (k = 9; tau^2 estimator: REML)

logLik deviance AIC BIC AICc

-21.6866 43.3731 47.3731 47.5320 49.7731

tau^2 (estimated amount of total heterogeneity): 9.2500 (SE = 6.7813)

tau (square root of estimated tau^2 value): 3.0414

I^2 (total heterogeneity / total variability): 73.04%

H^2 (total variability / sampling variability): 3.71

Test for Heterogeneity:

Q(df = 8) = 30.4571, p-val = 0.0002

Model Results:

estimate se zval pval ci.lb ci.ub

-3.2783 1.2355 -2.6534 0.0080 -5.6999 -0.8567 **

---

Signif. codes: 0 ‘***’ 0.001 ‘**’ 0.01 ‘*’ 0.05 ‘.’ 0.1 ‘ ’ 1

**Figure S3.** Forest plot of a between-group meta-analysis comparing PANSS general symptoms in schizophrenia patients receiving adjunctive raloxifene versus control, using a random-effects model with REML estimation.


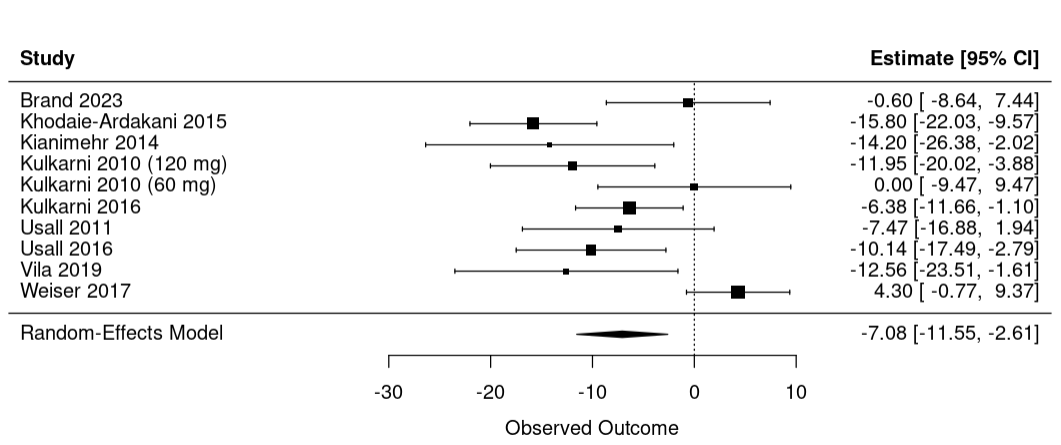


Random-Effects Model (k = 10; tau^2 estimator: REML)

logLik deviance AIC BIC AICc

-30.2730 60.5460 64.5460 64.9404 66.5460

tau^2 (estimated amount of total heterogeneity): 34.8454 (SE = 24.2595)

tau (square root of estimated tau^2 value): 5.9030

I^2 (total heterogeneity / total variability): 70.75%

H^2 (total variability / sampling variability): 3.42

Test for Heterogeneity:

Q(df = 9) = 35.1749, p-val < .0001

Model Results:

estimate se zval pval ci.lb ci.ub

-7.0775 2.2799 -3.1043 0.0019 -11.5460 -2.6090 **

---

Signif. codes: 0 ‘***’ 0.001 ‘**’ 0.01 ‘*’ 0.05 ‘.’ 0.1 ‘ ’ 1

**Figure S4.** Forest plot of a between-group meta-analysis comparing PANSS total symptoms in schizophrenia patients receiving adjunctive raloxifene versus control, using a random-effects model with REML estimation.
